# Whole genome sequence of *Klebsiella pneumoniae* U25, a hypermucoviscous, multidrug resistant, biofilm producing isolate from India

**DOI:** 10.1590/0074-02760150423

**Published:** 2016-02

**Authors:** Zumaana Rafiq, Nithin Sam, Rama Vaidyanathan

**Affiliations:** Dr MGR Educational and Research Institute, Department of Biotechnology, Chennai, Tamil Nadu, India

**Keywords:** *Klebsiella pneumoniae* genome sequence, multidrug resistant, carbapenem resistant OMPK36 porin mutant, India, CRISPR

## Abstract

*Klebsiella pneumoniae* U25 is a multidrug resistant strain isolated from a tertiary care hospital in Chennai, India. Here, we report the complete annotated genome sequence of strain U25 obtained using PacBio RSII. This is the first report of the whole genome of *K. pneumoniae*species from Chennai. It consists of a single circular chromosome of size 5,491,870-bp and two plasmids of size 211,813 and 172,619-bp. The genes associated with multidrug resistance were identified. The chromosome of U25 was found to have eight antibiotic resistant genes [*blaOXA-1*,*blaSHV-28*, *aac(6’)1b-cr*,*catB3*, *oqxAB*, *dfrA1*]. The plasmid pMGRU25-001 was found to have only one resistant gene (*catA1*) while plasmid pMGRU25-002 had 20 resistant genes [*strAB*, *aadA1*,*aac(6’)-Ib*, *aac(3)-IId*,*sul1,2*, *blaTEM-1A,1B*,*blaOXA-9*, *blaCTX-M-15*,*blaSHV-11*, *cmlA1*, *erm(B)*,*mph(A)*]. A mutation in the porin OmpK36 was identified which is likely to be associated with the intermediate resistance to carbapenems in the absence of carbapenemase genes. U25 is one of the few *K. pneumoniae*strains to harbour clustered regularly interspaced short palindromic repeats (CRISPR) systems. Two CRISPR arrays corresponding to Cas3 family helicase were identified in the genome. When compared to *K. pneumoniae*NTUHK2044, a transposase gene *InsH* of IS5-13 was found inserted.

*Klebsiella pneumoniae*, a Gram-negative, rod-shaped bacterium is an opportunistic pathogen that is commonly the cause of nosocomial infections. It is among the top five pathogens causing nosocomial infections worldwide (Rice 2010). Their multidrug resistant (MDR) nature poses serious healthcare issues. Over a period of 10 years, there has been an increase in the number of MDR *K. pneumoniae* resistant to cefotaxime, carbapenems, and piperacillin-tazobactum (Datta et al. 2012). Prevalence of fluoroquinolone resistant strains has also been noted in tertiary health care hospitals in Chennai, India (Magesh et al. 2011).

The strain *K. pneumoniae* U25 isolated from a urine sample has been studied extensively in our lab and was found to be a biofilm producer (Magesh et al. 2013). U25 was resistant to ciprofloxacin [minimal inhibitory concentration (MIC) 780 mg/L], PAβN sensitive (16-fold reduction of ciprofloxacin MIC on addition of 50 µM of efflux pump inhibitor PAβN) and showed enhanced intracellular norfloxacin accumulation in response to efflux pump inhibitor CCCP. This strain has also been used as a model to screen for efflux pump inhibitors from natural sources (Rafiq et al. 2016).

To study the bacteria at a molecular level, the genome of *K. pneumoniae*U25 was sequenced. Forty-three *K. pneumoniae* whole genomes have been deposited in National Center for Biotechnology Information (NCBI) genome database. This is the first report of the whole genome of *K. pneumoniae*species from Chennai.

DNA was extracted using Invitrogen DNA isolation kit and sequenced using PacBio single-molecule real-time (SMRT) technology (Eid et al. 2009). Geno- mic DNA extract was prepared as a 3-20-kb library for P6/C4 chemistry without Blue Pippin size selection. The PacBio RSII sequencing platform generated 90,559 reads, with a mean read length of 12,696-bp from one SMRT cell. The reads were assembled *de novo* with hierarchical genome assembly process 3 in SMRT Analysis v.2.3.0 (Chin et al. 2013). The best assembly was selected and circular contig was trimmed with Minimus 2 (Sommer et al. 2007). Base modifications were detected with SMRT analysis using default parameters.

The assembled U25 genome consists of a single circular chromosome and two plasmids. The genome size is 5,491,870 bp containing 57.38% GC and two plasmids of size 211,813 bp and 172,619 bp of GC content 52.4% and 52.6%, respectively. Other sequenced *K. pneumoniae* range from size 5,248,520 bp with GC content 57.7% (NTUH-K2044-NC_012731.1) to 5,641,239 bp with GC content 57.3% (KP 342-NC_011283.1). The genome was found to have 5,408 genes, 5,228 CDS, 64 pseudogenes, 25 rRNAs (5S, 16S, 23S), and 86 tRNAs. Two clustered regularly interspaced short palindromic repeats (CRISPR) arrays were identified at position 3,265,912-3,266,679 and position 3,275,432-3,276,010 which corresponds to CRISPR-associated Cas3 family helicase. In*K. pneumoniae* only a few strains harbour the CRISPR/Cas system. Apart from strain U25, only two complete *K. pneumoniae* genomes and four draft genomes sequences were found to harbour it. Studies have shown that in*Klebsiella* genomes it is located among genes encoding for proteins likely involved in metabolism and resistance to antibiotics (Ostria-Hernández et al. 2015). In U25, one CRISPR array with a 29 bp direct repeat had nine spacers and was located upstream the *cas3*gene. The other array with a 28 bp direct repeat had 12 spacers and was downstream*cas2*. The CRISPR system differed from those in *K. pneumoniae* NTUHK2044 in the number of spacers, and downstream of the CAS operon and the CRISPR repeats, a transposase gene InsH of IS5-13 was found inserted.

The genome was annotated using NCBI Prokaryotic Genome Annotation Pipeline (ncbi.nlm.nih.gov/genome/annotation_prok/) and confirmed using the Rapid Annotations using Subsystems Technology (Galens et al. 2011).

Phenotypically the strain U25 was found to be hypermucoviscous *in nature*and resistant to ciprofloxacin, norfloxacin, nalidixic acid, cefotaxime, cefoxitin, ceftazidime, amoxyclav, imipenem, and meropenem by antibiotic susceptibility test. The resistance genes were analysed by ResFinder-2.1 at Center for Genomic Epidemiology (CGE) server (cge.cbs.dtu.dk/services/ResFinder/). The genome contained two genes for cephalosporin resistance *blaOXA-1* and *blaSHV-28*, an*aac(6’)1b-cr* for quinolone and aminoglycoside resistance, a*catB3* gene for chloramphenicol resistance, *oqxAB*for quinolone resistance, and *dfrA1* for trimethoprim resistance in the U25 genome. Of these the *blaOXA-1* and the *aac(6’)1b-cr*are flanked by transposase of IS257 from Tn4003. The *oqxAB* operon along with the *oqxR* repressor and the *rarA* activator is in the chromosome and transposon elements are not observed around it.

The plasmid pMGRU2-P001 had only one phenicol resistance gene *catA1.*Plasmid pMGRU25-002 on the other hand, had the maximum resistance genes - aminoglycoside resistance [*strA*, *strB*,*aadA1*, *aac(6’)-Ib*, *aac(3)-IId*,*aadA1*, *aadA1*], sulphonamide resistance (*sul1*, *sul2*), beta-lactam resistance (*blaTEM-1A, blaOXA-9*, *blaCTX-M-15*,*blaSHV-11*, *blaTEM-1B*, *blaTEM-1A*,*blaOXA-9*), fluoroquinolone and aminoglycoside resistance [*aac(6’)Ib-cr*], phenicol resistance (*cmlA1*), macrolide resistance [*erm(B)*, *mph(A)*]. This plasmid is a multireplicon plasmid with two inc regions. As most resistance plasmids are conjugative and encode their own transfer, this might explain the accumulation of the antibiotic resistance genes (Bennett 2008).

Despite having two cephalosporin resistant genes in the genome and seven in the plasmid, the genes coding for carbapenemase enzyme was not found. Studies have shown that carbapenem resistance is also associated with a mutation in the outer membrane porin OMPK36 (Clancy et al. 2013). When the U25 genome was analysed, OMPK36 was found to have an insertion of glycine and aspartic acid at amino acid 134 (ins aa134-135 GD). Due to its location, this mutation can result in change of permeability and prevent antibiotics from entering the cell (Shi et al. 2013). This mutation would therefore account for the borderline resistance to imipenem. This is an important observation in a treatment point of view as a carbapenemase inhibitor would be ineffective in this case. This is the first report from India of a carbapenem resistant strain with the presence of a porin mutation in the absence of genes producing the carbapenemase enzyme.

The genome U25 was found to be very similar to genome PittNDM01-GCA_000733255.1. When analysed at the Multilocus Sequence Typing v.1.7 server of CGE (cge.cbs.dtu.dk/services/MLST/) (Larsen et al. 2012), both the genomes were found to belong to ST 14. They also had the same GC content of 57.4%. Genome alignment by mauve showed that they were very similar with only one area of inversion. Both genomes also contained the same antibiotic resistance genes and the same OMPK36 mutation. In addition, the PittNDM01 plasmid carries the*bla*NDM-1 and *blaOXA*-232 genes.

Therefore in this paper we report the first whole genome sequence of a *K. pneumoniae* species from Chennai. The antibiotic resistant genes were found essentially in one of the plasmids confirming the role of conjugative plasmids in spread of antibiotic resistance. Strain U25 was one of the few *K. pneumoniae*genomes containing CRISPR arrays which defend against entry of foreign DNA. The presence of an OMPK36 porin mutation likely to cause carbapenem resistance even in the absence of carbapenemase enzyme was identified. Strain U25, due to its hypermucoviscosity, MDR nature, and a biofilm production with efflux activity, plays a role in the evolution of highly resistant MDR isolates. The availability of this genome sequence will be valuable for comparative genomic analysis and in understanding the evolution of antibiotic resistance.

*Nucleotide sequence accession* - The *K. pneumoniae* U25 genome sequence was deposited in GenBank *via* Whole Genome Shotgun under the accession CP012043. The plasmid mMGRU25-001 was assigned the accession KT203286 and plasmid pMGRU25-002-KT818627.
